# Spectacles and Asthenopia

**Published:** 1897-07-10

**Authors:** 


### SPECTACLES AND ASTHENOPIA.

There are certain facts with which many of us are familiar enough, which, nevertheless, will bear repetition, and among them may be placed one to which Dr. St. John Roosa has lately called attention, viz., that asthenopia may be but an early symptom of neurasthenia. A point of interest on which Dr. Roosa insists is that it is not every case of hypermetropia, even when combined with a certain amount of astigmatism, which should be corrected by glasses.

The now well-known fact that headaches are often associated with defects in refraction has undoubtedly led physicians to transfer many cases to ophthalmic surgeons which they had far better have kept to themselves. Among students and over-worked clerks, and among operatives in textile factories, whose eyes are constantly engaged in watching trembling threads, headaches are very common, and there is a strong temptation, when hypermetropia is discovered in such cases, to look to glasses as the proper treatment. There is an even stronger temptation when their use relieves the headache to think that in ordering them the right thing has been done.

One may, however, have only put off the evil day, and, by giving to the patient a means of continuing the work which is doing him the harm, one may only be precipitating the catastrophe. The fact is, that a moderate amount of refractive defect ought not to cause asthenopia; and in those cases where it does so there is something the matter with the patient besides what is the matter with his eyes. Dr. Roosa says: "The greater part of the human race which is not myopic is hypermetropic. It cannot be said that a low degree of hypermetropia is, of itself, a sufficient cause of asthenopia. Just so with corneal astigmatism. Unless it reaches a diopter with the rule, or a quarter of a diopter against the rule, it does not of itself produce asthenopia." When in such cases he has met with asthenopia, he has repeatedly found it to be the precursor of nervous breakdown. To order glasses for such cases is not only a mere placebo, but may even be disastrous in its effects, in that it may veil the true condition of affairs and encourage the patients to think of their eyes as the cause of all their troubles.

We are all familiar with the desire for glasses so often expressed by patients recovering from severe illnesses, the asthenopia in this case being a mere sign of debility; and Dr. Roosa points out the interesting fact that malaria may cause the same condition. But he especially insists that the first sign of nervous breakdown may be an inability to use the eyes continually. However important, then, it may be to recognise the existence of a defect in the refractive power of an eye, it is still more important to discover why such an eye, which, notwithstanding that defect had previously served the patient well, has at last failed in its function.

